# How to Test for Seed Mucilage to Examine an Age‐Old Question: A Response to Ladwig and Lucas (2024)

**DOI:** 10.1002/pei3.70057

**Published:** 2025-06-05

**Authors:** E. F. LoPresti, J. M. Cowley, S. N. Gorb, A. Kreitschitz

**Affiliations:** ^1^ University of South Carolina Columbia South Carolina USA; ^2^ School of Agriculture, Food, and Wine University of Adelaide Adelaide South Australia Australia; ^3^ Kiel University Zoological Institute Kiel University Kiel Germany; ^4^ University of Wroclaw Wroclaw Poland

## Abstract

Traits of seeds are far less‐studied than those of vegetative plants, despite the importance of this stage in a plant's life cycle. Much research has demonstrated the importance of certain aspects of seed phenotype, including both chemical and physical traits, to survival in the face of biotic and abiotic selective pressures. One trait with demonstrated physiological and defensive functionality is seed mucilage. This persistent hydrogel coating on the surface of the seed is extremely common and found in thousands of species across angiosperms, with many independent evolutionary origins. Despite attention in taxonomic, floristic, ecological, and biomaterial investigations for over a century, and the economic importance of products derived from this mucilage, the trait is often overlooked, and protocols for the labs determining seed mucilage across plants vary. Here, in response to a paper claiming seed mucilage in many new species due to flawed methodology, we lay out specific protocols to determine the presence of mucilage, in an effort to standardize across studies. We hope these methods prove useful in both evaluating the current literature and permit cross‐study comparisons to advance the study of this important trait.

1

All seeds and fruits contain carbohydrates in various forms. Some also have a persistent, insoluble, mucilage envelope that surrounds the diaspore (hereafter: seed) when wetted (Figures 1, 2, 3). This functionally important hydrogel—characterized by its persistent viscoelastic physical properties—is both common across plants, important, and understudied (Grubert 1981; Western 2012). As a group of scientists interested in seed mucilage and its myriad ecological effects, we applaud Ladwig and Lucas (2024) for taking up this topic and examining it. Since Meinhardt Grubert's (1974, 1981) pioneering and comprehensive work, many research groups worldwide have taken up the topic for short periods of time, though it is rarely taken up as the full focus of any one research program. While especially insightful ecological work has been done by Tamara Western, Meike Englebrecht, Yitzchak Gutterman, Rachel Burton, Olof Ryding, and their colleagues (e.g., Gutterman and Shem‐Tov 1997; Ryding 2001; Western 2012; Engelbrecht et al. 2014; Phan and Burton 2018), this occasional attention over a half century has produced an extremely scattered literature. Therefore, we have just scratched the surface in both identifying the species with the trait, identifying the trait's myriad functions—including effects on germination, DNA repair, resistance to erosive forces, and defenses against granivores—and the selective pressures leading to the remarkable convergence of this trait across families and tissue types (a variety of hydrophilic carbohydrates in seed coat, fruit coat, and accessory fruit coats). Given that this trait is found in thousands of species (Grubert 1974, 1981), including many crops and agriculturally important weeds, much more deserves to be done.

**FIGURE 1:**
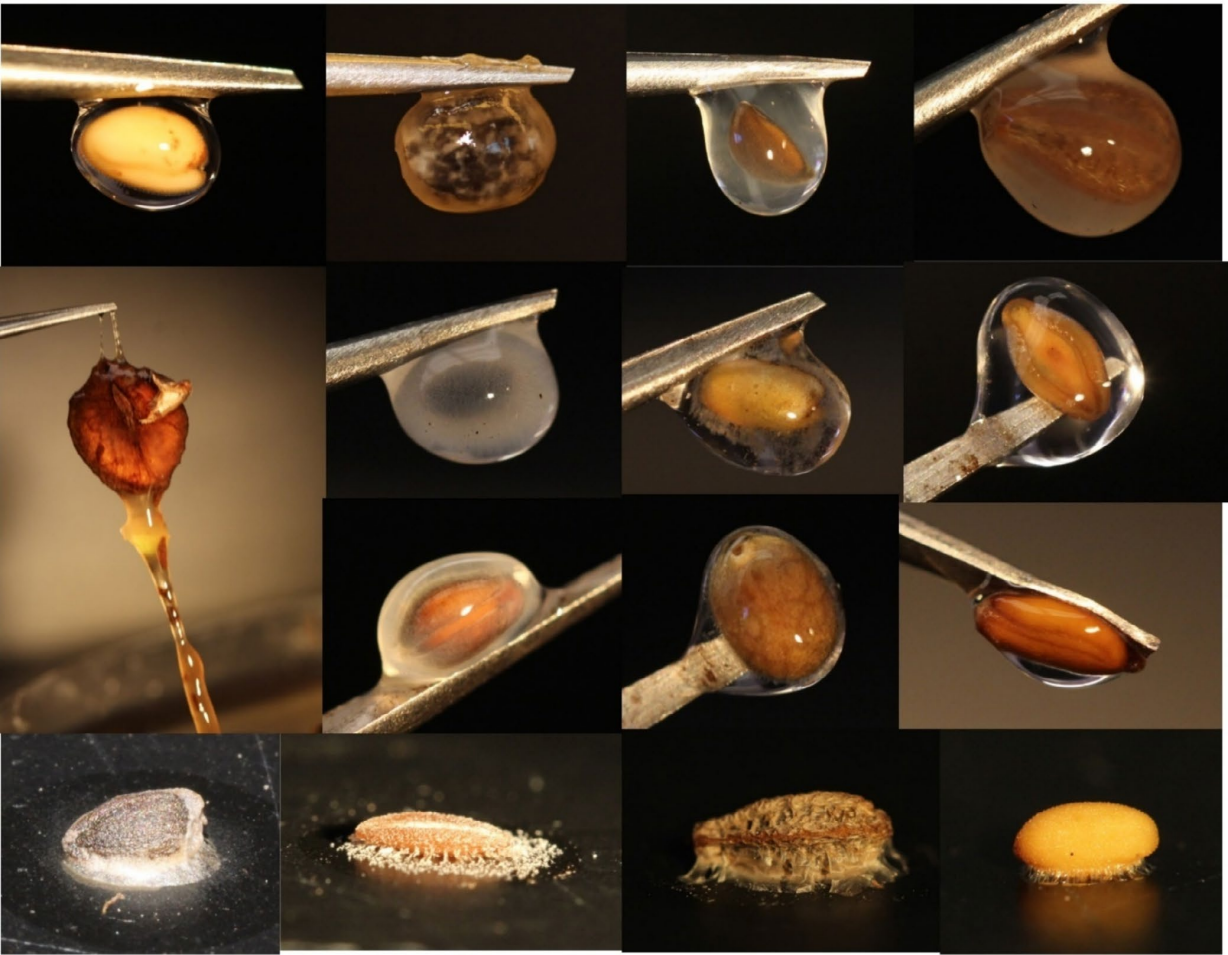
Examples of imbibed, dried, and stained imbibed seed mucilage. Imbibed mucilage—top row, left to right*: Camelina sativa* (Brassicaceae), *Croton capitatus* (Euphorbiaceae), *Dracocephalum moldavicum* (Lamiaceae), *Mirabilis nyctaginea* (Nyctaginaceae). Second row (L‐R), *Cobaea scandens* (Polemoniaceae), *Ocimum americanum* (Lamiaceae), *Plantago aristata* & *P. erecta* (Plantaginaceae). Third row (L‐R): *C. scandens* (again), *Prunella vulgaris* (Lamiaceae), *Salvia azurea* (Lamiaceae), *Stanleya pinnata* (Brassicaceae). Dried mucilage strands—fourth row (L‐R): *D. moldavicum, P. erecta, M. nyctaginea, C* *. sativa* .

In that vein, Ladwig and Lucas take up one of the oldest questions in the ecology and evolution of seed mucilage: is this trait more prevalent in arid environments? A sense—lacking much evidence, as Ladwig and Lucas correctly point out—is that arid areas have more species with mucilaginous diaspores. This idea seems to have originated in Murbeck 1919 (in Grubert 1974) and has been repeated ever since. Despite excellent work on mucilage in many species in mesic environments, including Swabrick's (1971) compendium for British plants and comprehensive analysis of mints by Ryding (2001), this sense of aridity being important persists to the present day, with only narrow, nuanced, confirmatory evidence of any relationship between mucilage and aridity (i.e., Ryding 2001; Teixeira et al. 2020; Pan et al. 2021; Cowley et al. 2022). However, Ladwig and Lucas's title and main conclusion—that moisture and mucilage have no relationship—are unsupported by their study design and statistics. We suggest that their confirmation of mucilage was mostly due to false positives of a soluble carbohydrate fraction—not a viscoelastic hydrogel—in the presence of a strong surfactant. Therefore, they were not testing for a persistent mucilage layer on the seed coat, the ecologically important trait of their references and that of the long‐standing hypothesis that they rightfully question. Additionally, the findings of far higher rates of seed mucilage than in any other study (almost double) and the identification of this trait in entirely new groups of plants are explained entirely methodologically (which they do point out, yet do not explore) and are not in line with how others have defined mucilage, making cross‐study comparisons impossible. The questions of whether mucilage and moisture have any relationship and how strong that relationship really is remain open, and we strongly encourage more testing.

Anyone interested in seed mucilage can easily go to a grocery or garden store and purchase several dozen species with this trait to observe it easily (chia, basil, flax, psyllium, etc.)—soaking those seeds for an hour produces an obvious gelatinous mucilage layer, shown in Figure 1. However, deducing the presence of the trait in those species with small amounts of external mucilage (including many Euphorbiaceae and Brassicaceae—Figure 1: *Stanleya pinnata* ) is more difficult, and the field has been hampered by a lack of standardized methodology. Complicating this still is that the only reasonably large compendiums, those of Grubert (1974, 1981) also include species with a mucilage component inside the seed, though the tissue layer was explicitly noted in this reference (Yang et al. 2012 included many examples of internal mucilage in their shorter compilation). This distinction is not just pedantic—those species without an external mucilage layer obviously will not have the same mucilage‐mediated interaction between the seed and any external factors, whether biotic or abiotic, that are the focus of the functional studies on seed mucilage. Ladwig and Lucas include both a true mucilage layer (in few of their species) conflated with a soluble carbohydrate component (most of their species), and while noted in the appendix, these are not differentiated in their analyses and conclusions.

## 2 Methodological Issues, and a Starting Point for Future Investigations

**FIGURE 2:**
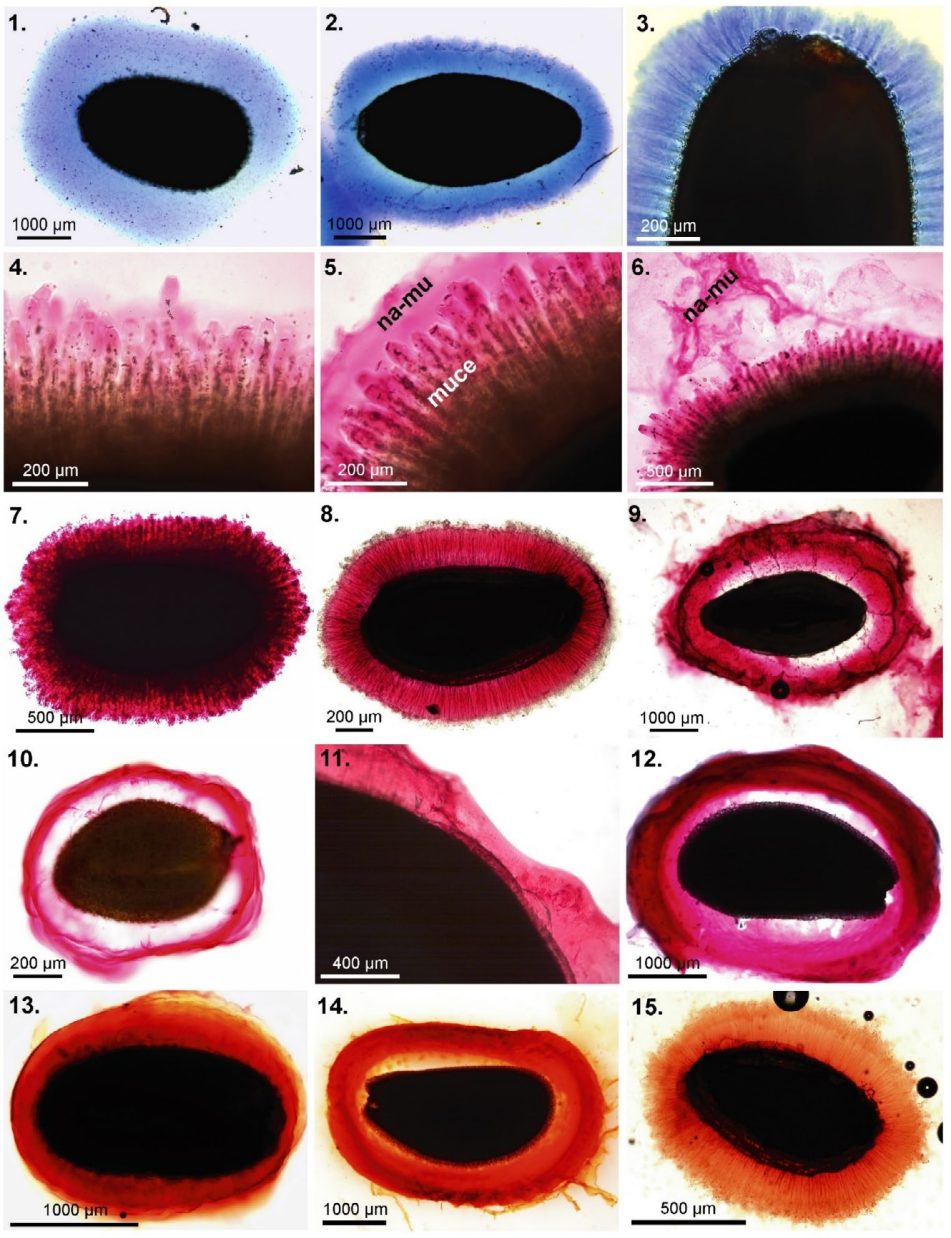
Staining of mucilage envelope components 1–3. Methylene blue (cellulose); 4–12. Ruthenium red (pectins); 13–15. Safranine (cellulose, pectins). 1. *Salvia hispanica* (Lamiaceae); 2. *Plantago indica* (Plantaginaceae); 3. *Capsella bursa‐pastoris* (Brassicaceae); 4–7. *Ocimum basilicum* (Lamiaceae), 4. Mucilage stained with ruthenium red (without earlier hydration) shows slowly formation of mucilage envelope. Within the mucilage envelope the swelling mucilaginous cells (muce) are visible and so is the slowly detaching, not dissolving, non‐adherent mucilage outer layer (na‐me) (5–6). After agitation or washing with water (7) this non‐adherent layer is not present anymore; 8. *Artemisia annua* (Asteraceae); 9. *Plantago ovata* (Plantaginaceae); 10. *Arabidopsis thaliana* (Brassicaceae); 11. *Linum usitatissimum* (Linaceae) with unevenly formed mucilage envelope; 12. *Lepidium sativum* (Brassicaceae); 13. *Capsella bursa‐pastoris* ; 14. *Lepidium sativum* ; 15. *Artemisia annua* .

**FIGURE 3:**
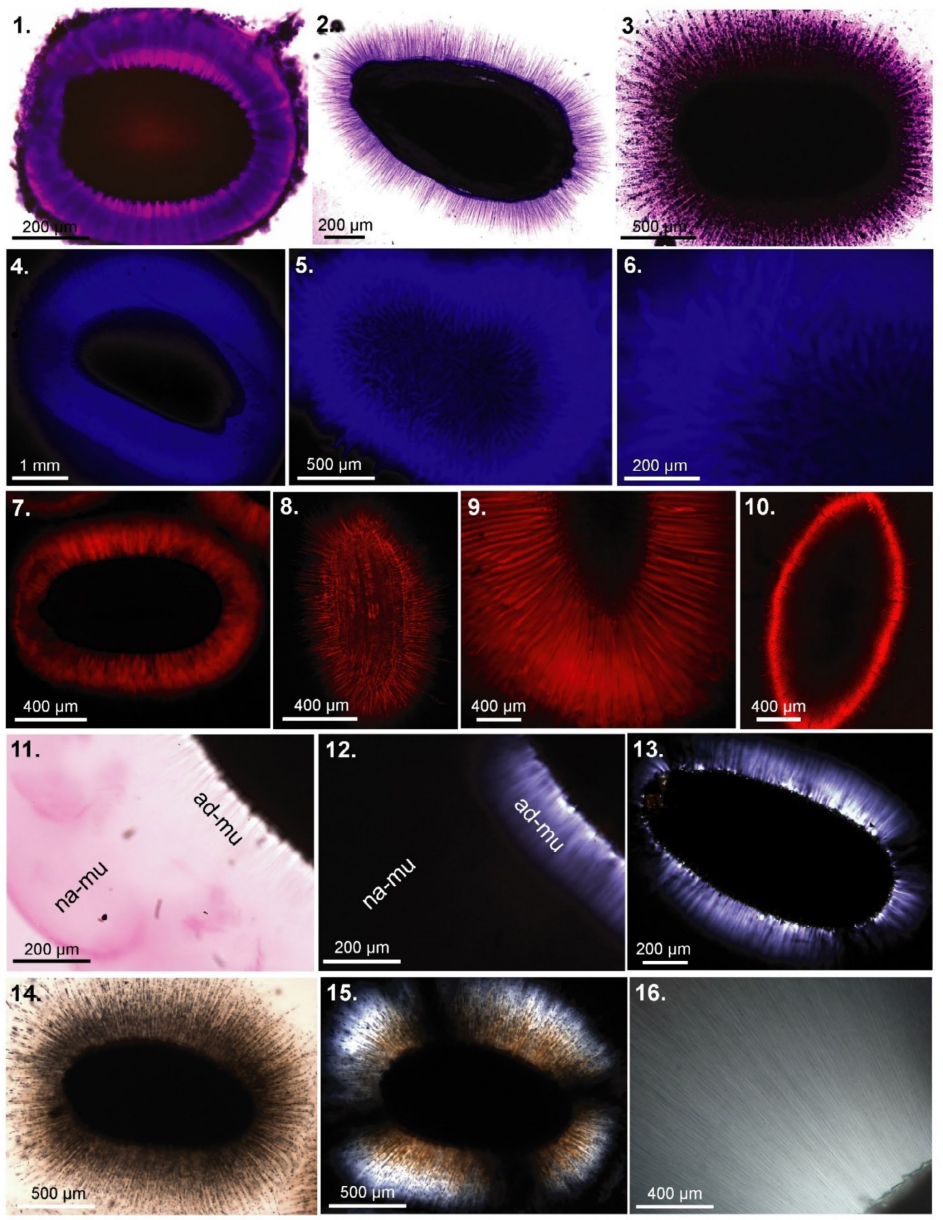
Staining of mucilage envelope components and detecting of crystalline cellulose. 1. Toluidine blue (pectins); 3. Crystal violet (cellulose); 4–6. Fluorescent brightener (calcofluor) (various β‐glucans, cellulose) ‐ fluorescence microscope; 7–10. Direct Red (cellulose specific) CLSM; 11. Ruthenium red; 12–13., 15–16. Crystalline cellulose—polarization microscope; 14. Distilled water. 1. *Arabidopsis thaliana* (Brassicaceae); 2. *Artemisia annua* (Asteraceae); 3. *Ocimum basilicum* (Lamiaceae); 4. *Lepidium sativum* (Brassicaceae); 5.‐6. *Ocimum basilicum* (Brassicaceae); 7. *Arabidopsis thaliana* ; 8. *Artemisia annua* ; 9. *Lepidium sativum* ; 10. *Plantago indica* (Plantaginaceae)—cellulose present in the remnants of the mucilaginous cells attached to the seed surface; 11. *Capsella bursa‐pastoris* (Brassicaceae), ruthenium red—visible non‐adherent mucilage (na‐mu) and inner adherent mucilage (ad‐mu); 12. The same as 11 but visualized in the polarization microscope. Inner layer of adherent mucilage with lightening cellulose fibrils (crystalline cellulose); 13. *Capsella bursa‐pastoris* —polarization of cellulose fibrils; 14–15. *Ocimum basilicum* with mucilage envelope formed after hydration in water (14) and visible in the polarization microscope (15); 16. *Salvia hispanica* —cellulose fibrils lightening in the polarization microscope.

**FIGURE 4:**
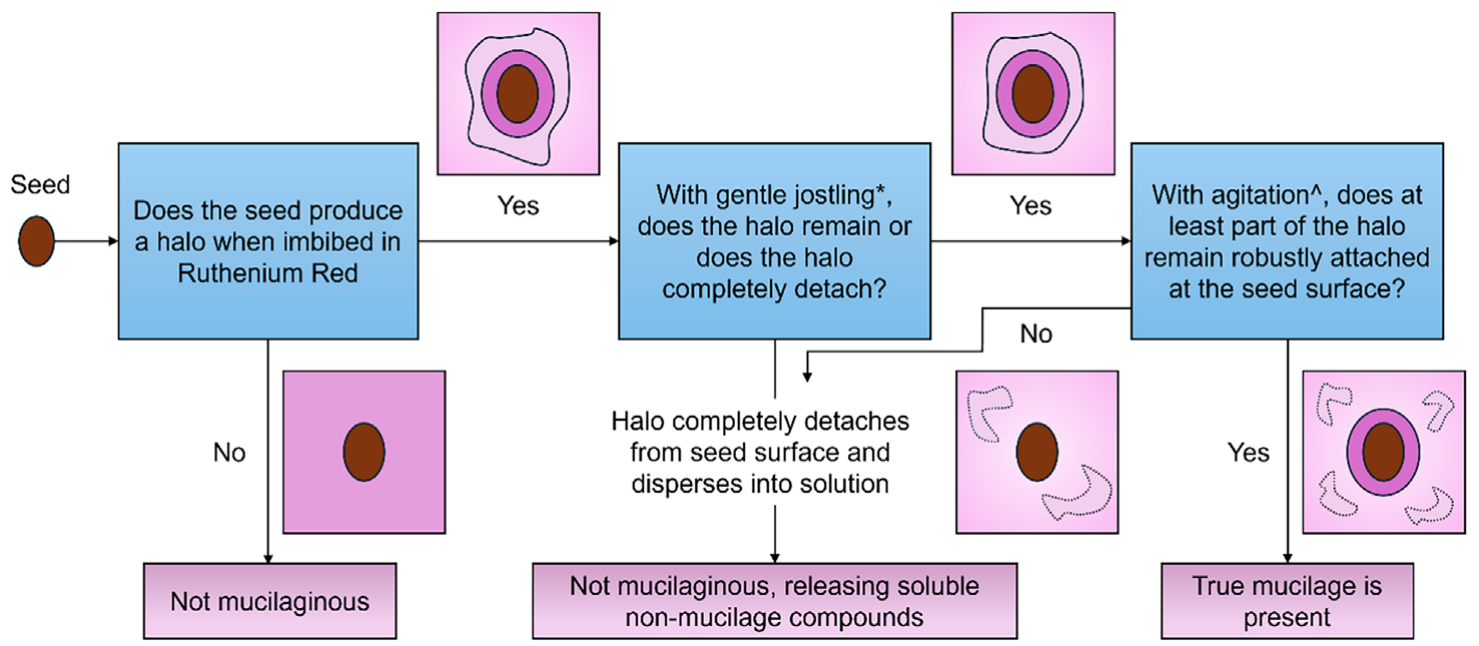
A conceptual diagram of the staining procedure for verification of mucilage when a simple soaking is insufficient. *A gentle tapping of the slide or plate that the seed and stain are in can be done simply to the stage of the microscope. ^This agitation might need to be fairly intense in order to remove the outer halo (i.e., true non‐adherent mucilage). For *Arabidopsis thaliana*, putting samples at 200 rpm in an orbital shaker for 30 min removes the non‐adherent layer.

All authors of this piece, who have tested tens of thousands of seeds, agree that in Ladwig and Lucas's Figure 1, of the six species they indicated had seed mucilage, only one— *Euphorbia corollata* —actually showed evidence of seed mucilage in the form of an envelope of insoluble mucilage around the seed (i.e., our Figures 1, 2, 3, 4). The others are all false positives, a stained solution, not mucilage layer around the seed, due to a soluble fraction of carbohydrates. They refer to this as “non‐adherent” mucilage in Table S1, accounting for 25 of their 37 positives, and while the term “non‐adherent” has been used in the literature previously for a component of the mucilage that is not strongly anchored to the seed (see Figure 4), we can find no instances of it used for a wholly soluble released substance not remaining in contact with the seed or inner envelope in any way. Therefore, their results are incomparable to those studies they note in Table 1 and, more importantly, not indicative of the conditions under which seed mucilage would function in nature. Though we all agree on what mucilage is, and what species have it, each of the authors here uses a slightly different protocol to ascertain mucilage, each honed independently over years of testing (and adjusted for different species). Therefore, the field is in need of a baseline way to begin tests to ascertain the presence of mucilage (note: Miart et al. 2018 lay out a good protocol that will work for most seeds, though it is more labor‐intensive than ours, and we disagree with a couple aspects). Given the false positives included with Ladwig and Lucas's methodology, we therefore suggest the following guidelines be a starting point for future investigations. Species‐specific differences in mucilage composition and density mean that one exact protocol is inappropriate—indeed, the first author's lab does two tests for each species, visualizing the mucilage both wetted and dried (Figure 1) and stains only when necessary to verify, where the latter three authors do staining to visualize the mucilage and structure first.

The goal of this protocol is to ascertain the presence of mucilage as well as visualize it; staining is often unnecessary to simply confirm mucilage—a basic wetting, as shown in Figure 1, is sufficient—but it also allows visualization of the structure of the mucilage envelope, which can be very different across species (Figures 2, 3). The stain ruthenium red is, indeed, the norm for staining mucilage and that which we all use, though methylene blue or toluidine blue also function well for some species (Miart et al. 2018, Figures 2, 3). While seed mucilage is highly variable in composition, ruthenium red importantly seems to be an extremely reliable diagnostic stain for released seed mucilage. However, as Ladwig and Lucas's photos show, it dyes certain other compounds as well—a property that is useful, but may be misleading. Ruthenium red is typically noted as staining specifically for pectic polysaccharides, however, not all mucilage is rich in pectin. Indeed, ruthenium red is also an excellent stain in species where pectin is not the predominant polysaccharide. In *Plantago ovata* (< 10% pectin), ruthenium red stains mucilage layers well including those where pectin is not present and in chia where true pectin domains are minimal or not present, other acidic residues are sufficient for good staining of the mucilage layer (Cowley et al. 2020, 2021; Ang et al. 2023). In summary, Ladwig and Lucas's selection of ruthenium red as a diagnostic stain for mucilage of potentially varied types is sound; however, the scoring of stained soluble components led to false positives (i.e., the soluble components shown in their Figure 1 a‐c,e are not seed mucilage).

The period of soaking or staining is also imperative—Ladwig and Lucas state that mucilage extrusion can occur over 24 h after seed hydration, without reference, but we have never seen evidence of such a long period in our own testing. Indeed, the functional trait of “seed mucilage” is that of a layer or envelope rupturing quickly from the seed coat or seed coat‐derived structure—we each use ~1 h, though some species may take as little as 30 s to imbibe (in *Euphorbia* sect. Anisophyllum it seems almost instantaneous) and even the few that require greater than an hour to fully imbibe will have a nearly full volume mucilage envelope long before that (LoPresti, pers. obs.; Pan et al. 2022). The prolonged soaking period that Ladwig and Lucas used probably allows non‐external polysaccharides to escape the seed, likely through the hilum (seemingly evidenced by the staining pattern in Figure 1C). In experiments where barley (*Hordeum vulgare;* a completely non‐mucilage producing species) grains are soaked for 24 h and then stained with ruthenium red, they do indeed show a slight acidic envelope that is not present at typical mucilage timescales (usually ~1 h) or even at 6 h imbibition times (Cowley, unpublished results). This layer is either microbial contamination or leached hydrophilic polysaccharides like pectin or mixed linkage glucan from the prolonged soaking (in Fabaceae, this is often galactomannan leached from the endosperm, Kreitschitz & Gorb, pers. obs.; also see the Fabaceae section in Grubert 1981). The entire argument of the authors is predicated on the functional definition—tested in each of their references—that the mucilage layer permits adherence, preserves water around the seed, allows dispersal, or enhances plant defense, none of which are likely with a soluble component that would not remain in contact with the seed. While they did find some species with the seed mucilage (nicely illustrated for *Euphorbia corollata* in their plate, a previously unreported species and a useful addition to the literature), the presence of a stained solution is not evidence for seed mucilage. It is also possible that some of the stained substance in the photos was residue attached to the outside of the seeds or included in seed packets.

Secondly, the author's exclusive use of EDTA to promote mucilage extrusion and study a naturally occurring phenomenon is highly problematic and surely explains some of the soluble carbohydrates stained and scored as mucilage in their tabulation. EDTA chelates Ca^2+^ ions in solution, which prevents the cross‐linking of pectin gels. Molecular biologists using *Arabidopsis thaliana* mucilage as a model system manipulate the availability of Ca^2+^ ions to examine differences in cross‐linking caused by manipulation of pectin chemistry and structure. In some examples, like the mutant line *fly1*, where cross‐linking is increased and mucilage extrusion is reduced, the use of EDTA restores a wildtype‐like mucilage phenotype (Voiniciuc et al. 2013). In more extreme phenotypes, *per36 and sbt1.7* mutants are unable to rupture the seed coat and release mucilage under normal conditions. Treatment of these mutants with EDTA enables cell wall loosening, rupture of the mucilage secretory cells, and normal mucilage release (Rautengarten et al. 2008; Kunieda et al. 2013). Importantly, natural accessions of *Arabidopsis* that are defective in mucilage extrusion and do not release it even after 48 h of soaking do so on treatment with EDTA, demonstrating that EDTA's treatment effect does not reflect natural conditions (Saez‐Agauayo et al. 2014). Furthermore, *Plantago* species that do not naturally release mucilage in water do so when treated with EDTA (James Cowley, unpublished results). It is also important to note that enhancing the presence of Ca^2+^ ions (e.g., by imbibing seeds in CaCl_2_ rather than water or EDTA) inhibits mucilage release through the opposite mechanism to EDTA (Voiniciuc et al. 2013). Due to the ubiquitous presence of calcium minerals in soil, one could argue that this would be more naturally relevant than EDTA treatment. Therefore, any results with EDTA, but not also tested by soaking in regular water, should not be regarded as accurately depicting natural conditions of mucilage release.

The Ladwig and Lucas paper, and the methods therein, made us each realize that the literature lacked a standardized methodology for mucilage detection and visualization. Hence, we would like to lay out our basic guidelines for testing. Despite all mucilage being a persistent hydrogel around the seed, the chemistry and quantity of mucilage differ greatly across species (Phan and Burton 2018) and for that reason, especially for effective visualization, individual species may be visualized somewhat differently.

## 3 Guidelines for Confirmation and Visualization of Mucilage Structure

### 3.1 A Visual Test for Obvious Mucilage

Before any staining, it is useful to simply soak the seed in deionized water for an hour, remove it from water with forceps, gently dab it dry on a towel, and examine whether there is any mucilage layer. An obvious mucilage layer—as you can see in unstained samples in Figure 1—is clear confirmation. However, in some cases, it is difficult to ascertain whether a little bit of mucilage is present, or whether a shiny surface is merely water (in Figure 1, *Stanleya pinnata* shows some of both). Practically, this method is also useful to demonstrate the effectiveness of mucilage removal methods (Cowley et al. 2025). A simple follow‐up—first done by Grubert 1974—is to then take those imbibed seeds and let them dry on a glass slide. The mucilage strands dry between the slide and the seed, cementing it in place (Grubert 1974; Pan et al. 2021, 2022; Stessman et al. 2023). For many species, those dried mucilage strands from the seed coat to the surface of the slide are obvious even without microscopy (Figure 1), for others, looking at the top and bottom of the slide under magnification can be useful. Grubert (1974) tested the adhesion by turning the slide vertically, blowing air on the slides, and “knocking the glass plate (in a vertical position) against the table”. For a very few species, surely < 5% overall that we have tested, these methods do not unambiguously settle the question of whether a species has mucilage. In those cases—and for any species in which you are interested in visualizing the structure of the mucilage—staining of the mucilage is the next step.

## 4 Staining for Visualization of the Mucilage Layer

Staining to see the mucilage layer is usually quick and straightforward, with the basic protocol laid out in Figure 4. The only required stain is Ruthenium red, which is available powdered or in solution from many scientific suppliers. These guidelines may need to be adjusted to nicely visualize the mucilage (i.e., Figures 2 and 3), but will suffice to demonstrate presence for most seeds with few adjustments.

1. Prepare the stain. Ruthenium red working solution (e.g., 0.01% w/w) should be relatively fresh and stored in complete darkness at +4°C to avoid oxidation. The stock solution (e.g., 0.1% w/w) will also be relatively stable at these conditions (~3 months). Partially oxidized ruthenium red solution is purple compared to a vibrant deep pink color, and it will have reduced staining efficiency. An entirely oxidized ruthenium red solution will form insoluble particles, which obscure imaging and do not stain.
2. Prepare and stain the seeds. Seeds should be cleaned of all debris, especially if field collected, and can be pre‐hydrated in water, briefly (< 30 min for most seeds), or imbibed directly in ruthenium red solution depending on the purpose. Doing both may allow a meaningful comparison of mucilage layers in some species. Pre‐hydration in water will remove any external soluble carbohydrates while direct imbibition retains some of that structure, if no strong agitation occurs. With certain mucilage layers, direct placement in a staining solution leads to excessively dark staining, which obscures mucilage features and even, occasionally, the seed margins themselves. This can be avoided with pre‐hydration or by immersing the overly stained seed in distilled water until it lightens up somewhat. Use of CaCl_2_ and EDTA solutions should be totally avoided to reflect natural imbibition conditions more closely.
  Mucilage expansion is non‐linear; a rapid expansion occurs on initial imbibition, with mucilage volume reaching ~90% of maximum after 30 min, with a much slower expansion in the several hours after (Phan et al. 2020; in careful tests of dozens of species, the longest time for any complete imbibition seed was 5.5 h for *Linum grandiflorum* , but it had a large envelope within an hour: (Pan et al. 2022). We each have settled on one hour for hydration (either with or without staining) without any issue and recommend the same to standardize across species and studies.
  During staining or pre‐hydration, seeds should be fully immersed in water without contact with air. Small seeds that do not break the surface tension should be stirred gently occasionally; those remaining in contact with the top of the water will have only partial imbibition of mucilage. Additionally, for small seeds (such as *Arabidopsis*) dissolved gases may nucleate as bubbles at the seed surface and may prevent efficient imbibition/mucilage hydration. Degassing by sonication with or without vacuum works well, as does gentle stirring throughout the soaking, though occasionally one or two seeds will retain a bubble and not imbibe well.
  Too many or too large seeds will deplete the ruthenium red solution and reduce staining efficiency—though for most studies screening a few seeds of a single species or population at a time for confirmation or visualization, this problem will likely be avoided. If imaging directly in ruthenium red solution in a cell culture plate format, the depth of the stain should be adjusted according to seed height to avoid stain opacity obscuring key mucilage features. A practical alternative for small seeds is to stain a few seeds on a microscope slide where the gap between the cover slip and slide (to be filled with stain) is dependent on the seed height. For screening presence/absence in many species, such fine adjustment may not be necessary, but for imaging to examine the size and structure of the mucilage envelope, these considerations are immensely important.
3. Image or score the seeds. For simple confirmation, the presence of a stained layer external to the seed coat (as in Figure 2) is sufficient. Photographs of well‐stained mucilage envelopes are useful for structural comparisons, as well as for quantification of envelope thickness and shape e.g., Pan et al. (2022). For those with a weak layer, gently jostle the seed; doing so may dislodge any secreted compounds or any contaminants—from the seed or microbes—on the seed surface. After this agitation, if a halo remains on the surface—whether thin or thick—some mucilage is present.
4. Complementing ruthenium red staining with other staining can be done—to differentiate the cellulosic components, Direct Red (cellulose) or calcofluor (β‐glucans and cellulose) work well, (Figures 2, 3); (Kreitshitz 2012) can be informative for visualization of strand structure, embedded components, or layers within the envelope. For example, mucilage is visible under polarized light due to the presence of cellulose fibrils and other birefringent structures. Additionally, placing seeds on a clean glass plate and letting them dry allows visualization of the dried mucilage strands which adhere the seed to the surface (i.e., Grubert 1974; Pan et al. 2021, 2022; Stessman et al. 2023; Kreitschitz and Gorb 2018—Figure 1) and the attachment to a smooth surface is a strong demonstration of the functional importance of the mucilage.
  In one methodology by Miart et al. (2018), a 24 h staining time was implemented, but in this method, an agarose gel impregnated with toluidine blue stain was used instead of a ruthenium red solution. While time‐intensive, their method works well for certain species, especially flaxes (*Linum* spp.). It offers a convenient way to manage these extremely slippery mucilaginous seeds, which can be unwieldy to image and quantify under non‐gel conditions, and for species with mucilage structure that can be difficult to image under aqueous conditions. However, the agarose gel induces an osmotic pressure from water binding in the gel, significantly slowing water uptake by mucilage and, therefore, increasing hydration times, and potentially creating a non‐naturally relevant hydration environment. Furthermore, for many species, especially Brassicaceae, seed germination/radicle emergence and testa rupture occur in these time scales, which leads to the release of toluidine blue‐stainable compounds, leading to a false positive (James Cowley, pers. obs.). For typical short liquid staining methods, this is rarely an issue but may be under these longer timescales needed for Miart's methods with certain species (though likely very few).

## 5 Other Considerations in Comparing Mucilage Production Across Habitats

The lack of consistent external mucilage testing is not the only drawback of this study. The inference you can draw from any comparison depends on your sample. By not selecting a random selection, or a representative selection, of species across their dry and wet grasslands, and instead choosing a commercial seed mix of 35/36 species commercially available from each, means that even with perfect mucilage detection methodology, they can conclude that commercial seed mixes intended for dry and wet prairies do not differ in mucilage production. (The mix is advertised on the supplier's website as “a colorful show despite dry conditions”—this is an ornamental mix: no more, no less). Their later assignment of ordinal groups and analysis via a categorical test does not improve inference given the same sample. Any further generality is unwarranted, and any analysis should have had a phylogenetic covariate—the greater selection of a family with seed mucilage in one habitat could massively skew any results. Lastly, the titular interpretation of no effect is a classic assertion of the null hypothesis, a nonstatistical interpretation (you can never prove a null) that needs to be made cautiously and only with a strong case made for the completeness of sampling. Therefore, we do not recommend future authors take these conclusions, or the species assignments presented in the supplementary table, as fact given the methodological and statistical issues.

## 6 Conclusion

We do not wish to end on a negative note; the authors deserve credit for taking a century‐old question that still lacks a clear answer, though we have outlined clear limitations of their methodology that limit comparisons to other studies. We hope that the definitions, methodologies, and guidelines proposed here help the field move forward and encourage other folks to take up this—and other—questions related to the evolution and ecology of this trait.

## Conflicts of Interest

The authors declare no conflicts of interest.

## Data Availability

Data sharing is not applicable to this article as no new data were created or analyzed in this study.
